# The Perception of School Life From the Perspective of Popular and Rejected Students

**DOI:** 10.3389/fpsyg.2022.801611

**Published:** 2022-04-07

**Authors:** Karla Hrbackova, Zuzana Hrncirikova

**Affiliations:** Department of Pedagogical Sciences, Faculty of Humanities, Tomas Bata University, Zlín, Czechia

**Keywords:** rejected and popular students, perception of the school environment, interpersonal and intrapersonal attitudes, peer rejection, upper primary school children

## Abstract

The experience of peer rejection in the classroom, an environment in which students spend a large part of their time, is accompanied by a sense of social pain which can have a profound effect on self-perception and attitude toward the overall school environment. These attitudes can be subsequently reflected in the student’s behavior at school and in his/her school success. The research aims to identify differences in the perception of school life (interpersonal and intrapersonal) among rejected and popular upper-primary school students. For this purpose, the sociometric nomination method and a questionnaire measuring the student’s perception of the school environment were used. From a total of 1,625 students (aged 11–15) from 20 schools, 312 students with the status of popular (liked) and rejected (disliked) were included in the research. The multivariate analysis of covariance (with age and gender as covariates) results revealed no significant differences between the two contrasting groups (popular and rejected) in terms of the perception of school life (interpersonal and intrapersonal). The results of the research indicated a different perception of the school environment *within* the groups of rejected and popular students rather than differences *between* the groups. Both the rejected and popular students report contradictory attitudes toward school life. Half of the students indicated that they feel lonely at school and have no confidence in teachers, considering the school a place where they do not like to learn, where they are troubled and where they do not like to talk to their classmates. Perhaps counterintuitively, a larger number of popular students stated that they feel lonely than did the rejected students from the class. Although the results do not paint a very positive picture of perceptions of the school environment, this should be seen as an opportunity to develop active class work with a greater emphasis on strengthening collective trust in the school.

## Introduction

School is one of the most important contexts for socialization and self-actualization among adolescents. The child-student needs not only the leadership of adults, but interaction with his/her peers, which also contributes greatly to social–emotional development. It is important that adolescents perceive the classroom peer context as positive and safe, i.e., they should feel comfortable around their classmates, feel included in the group, and experience few conflicts in the class (Boor-Klip et al., 2016). Although the class peer group is not selected by the student, she/he must interact with group members on a daily basis (Mertens et al., 2021). Relations among peers develop progressively, with various peer groups evolving within a specific class hierarchy in which students take on the role of classmates. Peer group relations are fundamental in a child’s development, affecting both school engagement and student academic achievement (Hurtado, 2018). Adolescents who feel that they are part of a school community are more likely to perform better academically and are more motivated in school; they are also less likely to engage in risky and antisocial behavior (OECD, 2017). Unlike other peer groups, the typical feature of a school class is non-selectivity. All members are putatively equal, sharing the advantages and disadvantages of daily school life together with the same rights and obligations (Rubin et al., 2015). In addition to the educational aspects, belonging to a class is a part of the student’s social identity, with the class representing a social group that influences the socialization development of adolescents (Guan and So, 2016; Albarello et al., 2021). Since admission to a group of peers is generally valued by students, interactions within the collective greatly contribute to the development of specific social skills (Blaževic, 2016). If a learner is accepted, she/he develops a sense of satisfaction and confidence through which self-esteem is built (Kulik and Kozieł, 2020). The position attained by the teens within the group then becomes an important part of their identity.

Group identity can represent a transitional phase which is often an intermediate step in the development of individual identity (Lage-Gómez and Cremades-Andreu, 2021). The various interpersonal relationships and social processes that occur in the classroom and other social spaces in the school shape the overall quality of the school environment. How students perceive, experience, and evaluate this environment can be influenced by their interpersonal relationships along with the position of the student in the classroom (Zandvliet et al., 2014). Although it can be assumed that the perception of the school environment will vary depending on peer preferences, we have relatively limited knowledge regarding how this perception of the school environment differs between rejected and popular students. Still, it may be assumed that a greater number of failed interactions with peers may cause lower social involvement as well as increased anxiety related to future social interactions, which in later years may also act as a barrier to career opportunities and the forming of other relationships.

## Peer Preference and Peer Rejection

Peer preference is defined as the measure of the “like” or “dislike” of an individual or smaller group by a wider peer group. High peer preference has been defined by being liked by many peers and disliked by few; low peer preference is being liked by few peers and disliked by many. Peer preference differs from other peer constructs such as perceived popularity, which focuses on social dominance and prestige rather than affective likes/dislikes (Parkhurst and Hopmeyer, 1998). Peer preference is an important aspect of relationships among children. In recent years, research in a number of disciplines has focused on peer rejection as a specific social phenomenon involving adolescent peer groups (Horn, 2003).

Research over the past two decades has highlighted the importance of peer rejection for the concurrent and subsequent adjustment of children. Rejection is a social phenomenon in which the main actors are the rejected child and the peer group, with the peer group shown to play a significant role in establishing and maintaining the status of the rejected child. Rejected children experience more negative expectations, behaviors, and interpretations of their actions than do other children (Coie and Cillessen, 1993; Milich et al., 1998).

Educators consider situations in which peers in the classroom reject a particular student to be but one aspect of normal social relationship formation (Smith and Brain, 2000). During schooling, children may find themselves in both the role of the rejecter and the rejected (Leary, 2001; Williams and Zadro, 2001). In one study conducted at the outset of the millennium, approximately one-third of young people reported experiencing some type of peer rejection (Deater-Deckard, 2001).

Social peer rejection is characterized by the avoidance of one member by most other members of the group (Townsend et al., 1988). A rejected child often serves as the group’s scapegoat (a singular object of active bullying or ostracization) and is thus disliked or even hated by his or her peers. In contrast to the simple binary of rejected/popular, some authors suggest that social rejection by peers should be considered in terms of a continuum of dynamic social behavior ranging from full inclusion to complete exclusion (Leary, 2001; McDougall et al., 2001). The consequences of social rejection can be severe, and may be manifested immediately or only after a certain period of time, negatively impacting the psychological well-being of the rejected individual.

The negative consequences of peer rejection include severe psychological problems such as poor adjustment (Buhs and Ladd, 2001), low self-esteem (Storch et al., 2003), suicidal behavior (Paulson and Everall, 2001), criminal behavior (Miller-Johnson et al., 1999), drug use (Reinherz et al., 2000), and lack of social skills (Seng, 2001; Wolpaw, 2001). Further, the mere threat of social ostracism has been shown to increase depression and suicidal ideation in children and adolescents (DiFilippo and Overholser, 2000; Laible et al., 2000).

## Loneliness

Loneliness occurs most frequently during adolescence (Heinrich and Gullone, 2006) and is a common experience for children and adolescents (Parkhurst and Hopmeyer, 1998). A common feature of adolescence is that it carries the risk of perceived social isolation, a state which must be distinguished from objective isolation (Laursen and Hartl, 2013). At the beginning of adolescence, changes take place in what children expect from their peers and parents, with peer relationships growing in importance (Parkhurst and Hopmeyer, 1998). A particularly significant finding is that loneliness has been shown to be higher in early adolescence than in later adolescence (Ladd and Ettekal, 2013).

Loneliness in children is influenced by how they are accepted by their peers, whether they have friends at all, the duration and quality of their closest relationships, and whether they are maltreated among peers (Asher and Paquette, 2003). Parker and Asher (1993) found that children without best friends are lonelier than children who report having a best friend. Low acceptance by peers contributes to a higher level of loneliness, although this is not synonymous with the absence of a close friend. McWhirter et al. (2002) report that social relationships are important in adolescence, as further evidenced by the findings of Chipuer and Pretty (2000) that adolescents more often experience social rather than emotional loneliness, i.e., the absence of a close relationship which fulfills the need for emotional attachment (a parent, best friend). In contrast, social loneliness represents a deficiency within a wider group (such as a peer group), i.e., it depends on the whole collective, not on individuals (Weiss, cited in Chipuer and Pretty, 2000). The basic trend in adolescence is that with increasing social support from classmates, the need for support from parents decreases (Hombrados-Mendieta et al., 2012).

Adolescents with poor or no social networks reported as being lonelier due to the absence of a group of friends with whom to engage in various activities as well as the absence of close, intimate friendships (McWhirter et al., 2002). It follows that psychological well-being during adolescence is influenced by the quality of peer relationships, a claim made by Hall-Lande et al. (2007) following their finding that social isolation is associated with an increased risk of low self-esteem as well as suicide attempts.

Demir and Tarhan (2001) found that among adolescents between the ages of 12 and 14, members of the rejected group of respondents were the loneliest. At the same time, the researchers compared this group with three other designated adolescent populations: a popular group, a controversial group, and a neglected group. In general, unpopular adolescents were rejected, while on the contrary, popular adolescents were liked. The students designated as neglected were those whom other peers overlooked completely, i.e., they were considered neither popular nor unpopular. The controversial group of adolescents was characterized by the fact that they were reported as popular with some peers and unpopular with others. Along with the unpopular adolescents, greater loneliness was indicated in the controversial group as compared to the popular group and neglected group. The investigators also offer an interesting explanation for this finding: being popular with some peers and unpopular with others can cause confusion and discrepancy. The child is aware of the instability in their peer relationships, and this confusion may cause greater loneliness for the members of this group, especially if the peers who like the child do not belong to the same reference group as does the child in question (Demir and Tarhan, 2001).

Peer status (as expressed by a peer preference score) in the classroom can play a key role in shaping the student’s attitude toward the school environment. The attitude to school determines how the student feels in this environment, e.g., as empowering or threatening to them (Kraft and Mayeux, 2016). Attitudes can significantly influence learning outcomes, the motivation for further learning and its perceived value as well as, finally, personal satisfaction and/or economic success in future life (Olalekan, 2016).

Our study follows other investigations based on the assumption that social acceptance, a condition which elicits positive emotions, is associated with positive perceptions of school life, while social rejection may be associated with negative perceptions of school life. Previous research has shown that school satisfaction is related to class climate and social acceptance. It has been found that school satisfaction is not determined solely by individual characteristics, but also to a large degree by class settings and structures (Verkuyten and Thijs, 2002). Research also points to a relationship between positive emotions and student satisfaction with school (Froh et al., 2008; Bordwine and Huebner, 2010). At least one study shows gender as a predictor of perceived school life, with boys scoring lower on school satisfaction than girls (GCR, 2021). Yet somewhat in contrast to these results, other research shows that gender and year of study significantly influenced the explicit school satisfaction of students, but not implicit satisfaction. In accordance with Wilson’s Dual Attitude Model (Wilson et al., 2000), Tian et al. (2010) defined these two constructs of school satisfaction, with explicit satisfaction indicated in self-reports, and implicit satisfaction evidenced in automatic or unconscious processes. Further, implicit perceptions of school life may be more greatly influenced by social or personal factors rather than by individual factors (Tian et al., 2010).

## Research Aims

The research aims to determine how students with contrasting peer statuses perceive the school environment. The intention is to verify whether students who are rejected from the class group perceive the school environment more negatively than do their popular classmates. Taking into account gender and age, the aim of this study was to examine differences in perceptions of school life (interpersonal and intrapersonal) among upper-primary school children who experienced rejection from the class (i.e., with the peer status of a rejected student) and those who experience acceptance in the classroom (with peer status as a popular student). The research is based on the assumption that a contrasting peer status may be reflected in the perception of this environment, i.e., unpopular (rejected) students may perceive the school environment as more threatening than do popular classmates, and age and gender has no effect on this perception.

## Materials and Methods

### Participants

A representative sample of 1,625 students from 20 upper-primary schools (6th−9th grade) in the Czech Republic was selected for the research population. The selection was carried out randomly through a random number generator from all schools in the Czech Republic in the Register of Schools of the Ministry of Education, Youth and Sports. The sample included 849 boys and 776 girls aged 11−15 years (average age 13 years, *M* = 13.17, SD = 1.287). In the sample, 182 rejected students (11%), 130 popular students (8%), 27 controversial students (2%), 231 neglected students (14%), along with 1,055 (65%) other students were identified. Only students with the sociometric status of rejected (unpopular) and popular were included in the sample, for a total of *N* = 312 students. All other students were excluded from the analysis. The research focused on the attitudes of two groups of students in the school class with the contrasting peer status of popular and rejected (unpopular). Comprising the group of rejected students (*N* = 182), 63% boys and 37% girls were identified, with 60% of all these students in the lower grades (grades 6–7) and 40% in the upper grades of primary school (grades 8–9). In the popular group (*N* = 130), 52% boys and 48% girls were identified, with 72% of students in grades 6–7 and 28% in grades 8–9.

### Research Tools and Procedure

The questionnaire School is a Place … (see Tables 1 and 2) was used to determine how students feel in the school environment. The student’s social position (i.e., peer preference rate) in the classroom was determined using peer nominations as indicated in a sociometric-rating questionnaire.

**Table 1:** Intrapersonal response rates for rejected and popular students.

**School is a place…**	Peer status	% yes, rather yes	% no, rather not
*where I like to learn*	Rejected	50%	50%
	Popular	52%	48%
*where I feel important*	Rejected	56%	44%
	Popular	57%	43%
*where I am often nervous*	Rejected	38%	62%
	Popular	48%	52%
*where I am happy*	Rejected	55%	45%
	Popular	54%	46%
*where I do not like being talked about*	Rejected	45%	55%
	Popular	51%	49%

**Table 2:** Interpersonal response rates for rejected and popular students.

**School is a place…**	Peer status	% yes, rather yes	% no, rather not
*where can I turn to the teacher when I have a problem*.	Rejected	46%	54%
	Popular	51%	49%
*where I feel lonely*.	Rejected	53%	47%
	Popular	50%	50%
*where there is good fun during breaks*.	Rejected	47%	53%
	Popular	46%	54%
*where we like to talk to other classmates*.	Rejected	44%	56%
	Popular	47%	53%
*where I am troubled*.	Rejected	49%	51%
	Popular	44%	56%

The School is a Place … questionnaire was designed as an abbreviated version of the original Students’ Attitudes to School Life Questionnaire (Vojtova and Fucik, 2012) used in the previous researches (Hrbackova, 2018). The Czech version of the latter questionnaire was based on the Quality of School Life Scale—School Life Quality Questionnaire (Williams and Batten, 1981) as well as on the work of Binkley et al. (1996). The School is a Place … questionnaire contains 10 items designed to identify how students feel in the school environment on two levels: in relation to others, i.e., the interpersonal level (five items), and in relation to themselves, i.e., the intrapersonal level (five items). The answers are expressed on a Likert scale of 1 (definitely yes) to 4 (definitely not). The results are expressed by an overall score of a minimum of 5 to a maximum of 20 points. The mean values of the calculated score (*M* = 12.5 points) represent ambivalent attitudes, with the higher the score, the more negative the students perception of the school environment, and the lower the score, the more positive. Based on the principal component analysis, we have verified that both factors (interpersonal and intrapersonal) explain 50.17% of the variance. The interpersonal factor (1) explains 33.25% of the variance and includes five items with a weight factor of 0.63−0.88. This factor expresses how students feel in the school environment in relation to others, i.e., the student’s feelings of closeness or openness to others. A higher score (min. 5−max. 20) expresses a greater degree of negative feelings experienced in the school environment and thus a higher degree of isolation and loneliness toward others. A lower score reflects more positive feelings experienced in the school environment and a higher degree of openness to others. A positive direction indicates that the school environment serves as a strengthening factor, while a negative direction indicates that the school environment functions as a threatening factor. The intrapersonal factor (2) expresses the students’ feelings at school in relation to themselves. A higher score (min. 5 - max. 20) expresses a negative experience associated with the student’s perception of her/his own feelings in the school environment, with a lower score representing positive feelings experienced in the school environment. A positive direction suggests that the student perceives the school as an environment in which they feel good, i.e., the environment is perceived as empowering. In contrast, a negative direction suggests that the student perceives the school as an environment in which they do not feel comfortable or safe, i.e., they perceive this environment as threatening. This factor includes five items with a weight factor of 0.33−0.62 and explains 16.92% of the variance. Measured using Cronbach’s coefficient, the internal consistency of all 10 items in the questionnaire attains a value of *α* = 0.712. McDonald’s omega coefficient reaches *ω* = 0.735, representing a good measure of internal consistency (Cortina, 1993). The internal consistency for the intrapersonal factor reaches a value *α* = 0.504, *ω* = 0.513, and for the interpersonal factor reaches a value of *α* = 0.831, *ω* = 0.839, which represents an acceptable level of reliability. The structural model provides a good model fit with the following indices: *χ*^2^/*df* ratio = 0.918, *p* = 0.495; GFI = 0.949; RMR = 0.074; CFI = 0.941; RMSEA = 0.069; PCLOSE = 0.063.

To determine peer status, a sociometric-rating questionnaire was used. The most widely used method to measure sociometric status is peer nomination, through which the participants are asked to nominate peers they like the most or the least. The measurement of sociometric peer status is based on the peer nomination items “liking” (e.g., acceptance) and “disliking” (e.g., rejection) by which peer status was determined. Unlimited nominations were used, and self-nomination was not allowed. The students were asked “Whom do you like the most?” (LM) and “Whom do you like the least?” (LL). They were also instructed to nominate classmates through a best-friendship question (“Who are your three best friends?”) as well as through an acquaintanceship question (“Who do you hang around with?”). The LM and LL items were used to calculate a peer preference index for each student according to the procedure of Coie et al. (1982), with the raw nominations for LM and LL ratings tallied, standardized, and transformed into a peer preference score. The rejected group consists of all students who received a peer preference standardized score of less than −1.0, an LL standardized score of greater than 0, and an LM standardized score of less than 0. The popular group consists of all students who received a peer preference standardized score of greater than +1.0, an LL standardized score of less than 0, and an LM standardized score of greater than 0.

The data was collected from students during classes using paper−pencil assessment, with the students filling in the questionnaires based on the teacher’s instructions. The data was processed using the IBM SPSS program version 28. Firstly, descriptive statistical analysis was conducted, then to further analyze group differences the independent sample *t*-test and multivariate analysis of covariance (MANCOVA) was conducted (with age and gender as the covariates). The independent sample *t*-test was used to analyze the comparison of means regarding attitudes toward school of two independent groups (popular and rejected students). The MANCOVA was used to test the statistical significance of the effect of independent variable (peer status) on a set of two dependent variables (interpersonal and intrapersonal attitudes toward school life) after controlling for age and gender as covariates. In the MANCOVA analysis, Bonferroni alpha (0.05) corrections were used. To illustrate the results, simple frequency tables were used, with the results showing the prevailing distribution of attitudes toward school in the two contrasting peer groups (popular and rejected).

## Results

The results of the research in Table 3 show that the rejected and popular students perceive the school environment very similarly both at the intrapersonal level (*p* = 0.108) and interpersonal level (*p* = 0.470). At the intrapersonal level, the rejected students scored *M* = 11.73 points (SD = 3.098), the popular students *M* = 12.25 (SD = 2.345). At the interpersonal level, the two groups of students with different peer statuses also achieved comparable results, with the rejected students scoring *M* = 12.87 points (SD = 3.951) and the popular students *M* = 12.46 (SD = 5.138).

**Table 3:** Perception of the school environment in rejected and popular students.

	Peer preference	Mean	SD	Std.
Interpersonal	Rejected	12.87	3.951	0.302
	Popular	12.46	5.138	0.467
Intrapersonal	Rejected	11.73	3.098	0.239
	Popular	12.25	2.345	0.214

*MANCOVA (age and gender covariates) comparison of means scores showing no significant group differences: Wilks’s *Λ* = 0.985, *F*(2, 270) = 2.01, *p >* 0.05, *η^2^* = 0.015; gender, *p* = 0.651; age, *p* = 0.410*.

The MANCOVA (with age and gender as covariates) results also revealed no significant differences (*p* = 0.137) between the popular and rejected students with regard to interpersonal and intrapersonal attitudes toward school: Wilks’s *Λ* = 0.985, *F*(2, 270) = 2.01, *p* > 0.05, η^2^ = 0.015; gender, *p* = 0.651; age, *p* = 0.410.

On average, the perception of the school environment appears ambivalent in both groups (Table 3). Nevertheless, this result is due to the significant variance in the students’ responses (either positive or negative).

The response rates shown in Table 1 indicate a different perception of the school environment within both groups rather than a different perception between the two groups of rejected and popular students. The rejected students as well as their popular classmates indicated that they experience similar feelings in the school environment. What is striking is the high percentage of students who perceive the school environment negatively, regardless of whether they are rejected or accepted among classmates. A total of 50% of the rejected and 48% of the popular students showed a tendency to dislike learning at school. 46% of the popular and 45% of the rejected students indicated that they perceive school as a place where they are not happy; 45% of the rejected students and 51% of the popular students reported to not like being spoken about at school. 48% of the popular and 38% of the rejected students indicated feeling nervous at school, and 44% of the rejected and 43% of the popular students reported not feeling important at school.

The student’s perception of the school environment in relation to others (interpersonal) tends more toward negative values (on scale ranging from completely positive to completely negative perceptions) than in relation to themselves (intrapersonal). The frequency of responses in Table 2 shows that only 53% of the rejected students, but also 50% of the popular students indicated experiencing loneliness in the class. Similarly, communication with classmates was found to be unpopular with both groups, with 53% of the popular students from the class team reported not liking to talk to their classmates and 44% of rejected students from the class team indicating that they in fact like to talk to their classmates.

Partial responses show that 54% of rejected and 49% of the popular students indicated that they cannot turn to the teacher when there is a problem. 53% of the rejected and 54% of the popular students report that there is no fun during breaks, and 49% of the rejected students and 44% of popular students report that they suffer at school. These values are clearly unfavorable.

The differences among the responses of the rejected students from the class are also interesting. 50% of these students perceive school as a place in which they can learn. 56% of the rejected students feel important at school, and 55% of these students feel happy at school. 47% of the rejected students do not feel lonely at school, and 51% of these students do not perceive school as a place where they struggle. The rejected students even reported that “Breaks are good fun” (47% of students), and that they like to chat with their classmates (44% of students).

## Discussion and Conclusion

The research results show no significant differences in the perception of the school environment between peer-rejected students and their popular classmates. The conclusions do not support the assumption that students rejected from the classroom perceive the school environment more negatively than their popular classmates, i.e., these designated groups perceive the school environment similarly. These findings are not consistent with research showing that peer status has a greater impact on school perception than does friendship, e.g., Osterman (2000) has published findings showing that children who are accepted by their peers are more likely to their classes and school in general. Similarly, Huebner and McCullough (2000) found that school satisfaction is related to the students’ assessment of how they feel about this environment in relation to the importance of the school, the school community as well as the interpersonal relationships experienced in this context. The fact that peer status does not seem to affect the perception of the school environment may be due to a discrepancy between implicit and explicit attitudes, a view which is consistent with the Wilson’s Dual Attitude Model (Wilson et al., 2000). It is possible that rejected students outwardly show explicit attitudes, with implicit attitudes tending not to be shown (which is to be expected given that these attitudes are often unconscious). Martín-Antón et al. (2016) report that some children have a privileged social status: “they are the preferred students, highly valued by their peers” (p. 2). Others simply get on well with others and have several friends. But there are also children who for various reasons do not fit in and are passively or actively rejected and excluded by their peers. This peer rejection is associated with the experience of social pain (Eisenberger, 2013), which activates various coping strategies to deal with rejection from the class (as a threatening situation). One of these strategies may be expressive suppression. Based on research findings on expressive suppression and pain empathy (Anderson et al., 2021), expressive suppression of pain expression faces was found to reduce neural representations of negative emotion. According to Hart’s (2014) Integrative Theory of Psychological Defense, self-deluding defense mechanisms are primarily motivated by a sense of insecurity characterized by the experience of vulnerability and a lack of confidence in one’s own ability to cope with threats. Uncertainty can arise from various sources, such as attachment relationships, self-esteem, or conflicts in beliefs.

Our research shows that regardless of peer status students perceive school life in contradictory ways. Our findings carry a number of disappointing implications. About half of students (regardless of their peer status) do not consider school to be a place where they like to learn, and more than half of the students would not turn to the teacher if a problem occurred. Almost half of the students are troubled at school; they are not happy and they feel lonely. More than half of the students do not consider school to be a place where they like to talk to their classmates and they do not view breaks as a time of good fun. The conclusions of other research surveys in the Czech context correspond with our results. The feeling of belonging to the school has weakened among Czech students from 2003 to 2012 (OECD, 2014). In 2015, the index of sense of belonging was the lowest among OECD countries (OECD, 2019). PISA (OECD, 2019) shows that 30% of students in Czech schools involved in their survey experienced some form of bullying several times a month. The research also indicates a link to online behavior. Students who report spending more than 6 h a day on the internet (about 26% of students) feel more comfortable alone, and they have lower expectations of continuing their education than do students who spend less time online (OECD, 2017). The percentage who report feeling like an outsider at school has increased on average in many countries between 2003 and 2015 (OECD, 2017).

Our research results show a different perception of the school environment *within* groups of rejected and popular students rather than differences *between* the groups as taken separately. The results in the responses of students rejected from the class are encouraging, as more than half of the rejected students still experience a sense of importance. Similarly, almost half of the rejected pupils feel happy at school. Almost half of the rejected students claim that they enjoy school breaks, do not feel lonely at school, and like to chat with their classmates. It is possible that rejected students do not perceive the school environment as threatening when compared with other environments which they encounter.

The basic mechanism of social exclusion from the class is mainly due to student diversity, i.e., students become excluded because of their specific differences from others (Harrist and Bradley, 2002). Bauman (1998) has defined the term *symbolic social exclusion*, a situation associated with the stigmatization of individuals stemming from stereotyped perceptions of differences seen as disadvantageous. In this context, the group of students rejected from the class group often includes children from socially disadvantaged backgrounds. From an existential point of view, family functionality can be fundamentally reflected in the assessment of the importance of other relationships, including the school environment (Zandvliet et al., 2014).

We did not find peer status to play a role in the different perceptions of the school environment. The statements from our student respondents indicate that half of them, regardless of their peer status, perceive the school environment negatively, especially in relation to others. This was a surprising and unexpected result. Judged by the negative valence of the responses, the school environment (especially at the interpersonal level) may be perceived as threatening. The incongruities in the statements among the rejected students in the class seems interesting, with this ambiguity in the overall perception of the school environment leading us to several possible interpretations. It could be that rejected students are internally aware of their position in the classroom but outwardly deny this situation (a form of defense mechanism). Or they may fully acknowledge their situation and simply identify with it, which may largely be related to their level of self-esteem.

Our results suggest that regardless of social status, students generally perceive the school environment in very contradictory ways, a situation which does not provide a very satisfactory overall picture. These findings suggest that there is a need to identify the causes of pupil dissatisfaction toward school and take steps to alleviate the situation, e.g., by strengthening collective trust at school among all students (Forsyth et al., 2011). High collective trust can only be established in an environment which meets the student’s basic psychological needs—autonomy, healthy relationships, ways of developing competence (Deci and Ryan, 2008). In such surroundings, students can develop their own independence and abilities (autonomy), establish and maintain meaningful interpersonal connections, e.g., by belonging to a class (relationships), and gain confidence by feeling that they do something well (competence; Adams et al., 2015). Such an environment can be described as an self-regulatory climate, i.e., a multidimensional interconnected system that affects the quality of social relations and positively influences the dynamics of the classroom as well as other environments (Adams et al., 2016). Collective faculty trust in students, collective student trust in teachers as well as “student-perceived academic emphasis” are prerequisites for establishing and strengthening a self-regulatory climate (Forsyth et al., 2011). These conditions can be crucial for transforming the school environment so that students perceive it as empowering (rather than threatening). As a positive perception of the school environment is strengthened, social relations among all school actors in the class develop in affirmative and constructive ways.

Some limitations of this study should be noted. First, the measurement of perceptions of school life was very specifically targeted. Although other areas of school life could have been considered, the research focused on finding out how students felt in the classroom in terms of interpersonal and intrapersonal relationships. Second, the research focused on two groups of students (the rejected and popular students) because of the contrasting status of students in the classroom. The research did not focus on the perceptions of all students and did not consider other sociometric statuses in the classroom. The two contrasting groups can be more revealing about perceptions of school life. Third, the partial results are shown using simple descriptive statistics to highlight the prevalence of frequencies in the positive or negative direction. Finally, it should be emphasized that although the present study was based on an extensive research sample, the results may or may not prove generalizable beyond the Czech context.

In summary, two important findings can be emphasized. Firstly, the group of rejected students and the group of popular students were not uniform in their attitudes, thus problematizing the view that these two groups of students (whether they are the rejected student group or the popular student group) can be designated as homogeneous entities. Secondly, the attitudes expressed by the respondents toward school life cannot be explained by peer group preference. These outcomes highlight the need to work with the whole class together regardless of peer status.

## Data Availability Statement

The raw data supporting the conclusions of this article will be made available by the authors, without undue reservation.

## Ethics Statement

The studies involving human participants were reviewed and approved by Ethics Committee of Tomas Bata University in Zlín. Written informed consent to participate in this study was provided by the participants’ legal guardian/next of kin.

## Author Contributions

The authors confirm contribution to the paper as follows: KH: study conception and design, data collection, analysis and interpretation of results, and draft manuscript preparation. ZH: theoretical concept of the article and theoretical background. Both authors reviewed the results and approved the final version of the manuscript.

## Funding

This research was supported by the Czech Science Foundation GA CR 17-04816S *The Dynamics of Self-Regulation in Socially Excluded Students;* and Tomas Bata University in Zlín, Faculty of Humanities RO60211011025/2102.

## Conflict of Interest

The authors declare that the research was conducted in the absence of any commercial or financial relationships that could be construed as a potential conflict of interest.

## Publisher’s Note

All claims expressed in this article are solely those of the authors and do not necessarily represent those of their affiliated organizations, or those of the publisher, the editors and the reviewers. Any product that may be evaluated in this article, or claim that may be made by its manufacturer, is not guaranteed or endorsed by the publisher.
